# Intersections between patient-provider communication and antenatal anxiety in a public healthcare setting in Pakistan

**DOI:** 10.1371/journal.pone.0244671

**Published:** 2021-02-05

**Authors:** Asiya K. Kazi, Armaan A. Rowther, Najia Atif, Huma Nazir, Maria Atiq, Shaffaq Zulfiqar, Abid Malik, Pamela J. Surkan

**Affiliations:** 1 Department of International Health, Johns Hopkins Bloomberg School of Public Health, Baltimore, Maryland, United States of America; 2 Human Development Research Foundation, Islamabad, Pakistan; 3 Rawalpindi Medical University, Rawalpindi, Pakistan; Kwame Nkrumah University of Science and Technology, GHANA

## Abstract

This study explores pregnant women’s and healthcare providers’ perspectives on the role of patient-provider communication in experiences of antenatal anxiety within a low-resource setting. In 2017–18, we consecutively sampled pregnant women (*n* = 19) with at least mild anxiety and purposively sampled antenatal care providers (*n* = 10) from a public hospital in Punjab Province, Pakistan. We then conducted in-depth interviews and thematically coded them with a combination of inductive and deductive coding methodologies. We found that patients expressed a desire for warm, empathetic communication from providers who demonstrate respect, attentiveness, and a shared lived experience. Providers revealed an awareness that their heavy caseloads, high stress levels, and discourteous tones adversely influenced communication with pregnant women and may exacerbate their anxieties, but also reported that compassionately addressing women’s concerns, providing financial problem-solving and/or assistance, and moderating conflicting healthcare desires between patients and their families could alleviate anxiety in pregnant women. Patients reported feelings of anxiety stemming from a belief that they received lower quality communication from antenatal providers at public hospitals than patients received from antenatal providers at private hospitals, an experience that they partially attributed to their low socioeconomic status. Meanwhile, some providers disclosed potentially stigmatizing views of women from particular sociocultural backgrounds or low socioeconomic status, including perceptions that appeared to shape communication with these patients in antenatal care encounters. Our findings provide preliminary evidence that communication between pregnant women and antenatal providers that is warm, normalizes patient fears, and integrates patients’ interpersonal and financial considerations can mitigate pregnant women’s experiences of anxiety and reduce barriers to accessing antenatal care in Pakistan’s public healthcare facilities.

## Introduction

Symptoms of anxiety are characterized by excessive and uncontrollable worry that hinders daily functioning [1]. Anxiety during pregnancy is a common and concerning public health problem, with an estimated prevalence of 34% in low-and-middle income countries (LMICs) [2] and up to 49% in South Asia specifically [3, 4]. In comparison, antenatal maternal anxiety in upper-middle and high-income countries has been estimated to range from 18–29% [5–7]. One meta-analysis found that 21% of women worldwide have at least one anxiety disorder during pregnancy or the postpartum period [8]. For the child, antenatal maternal anxiety predicts reduced adaptive immunity in infants [9], reduced grey matter density in children aged 6–9 years [10], as well as internalizing behaviors and elevated cortisol levels for children at age 15 [11, 12]. Antenatal anxiety is associated with higher medical risk, including but not limited to gestational or pre-existing diabetes, vaginal spotting or bleeding, and preterm birth [13]. It is also associated with higher perceived risk of birth complications [13], higher risk of pregnancy-induced hypertension [14], and increased probability of undergoing a caesarean section [15].

Symptoms of anxiety can be heightened during pregnancy because of fears of adverse outcomes related to the delivery or the child’s health [4]. Maternal mortality occurs predominantly (94%) in low-resource settings [16]; moreover, the maternal mortality ratio (MMR) in low-resource settings is 4.62 per 1000 live births, greatly outnumbering the MMR of 0.11 per 1000 live births in high-income countries [17]. Thus, in LMICs such as Pakistan, where rates of maternal mortality (1.40 per 1000 livebirths [16]) and neonatal death (42 per 1000 live births [18]) are relatively high, fear of adverse birth-related outcomes may contribute to anxiety among pregnant women. Given the burden of antenatal anxiety in LMICs and the potential sequelae, it is important to examine how women’s healthcare experiences relate to their experiences of anxiety during the antenatal period.

Patient-provider communication refers to verbal and non-verbal communication that occurs between a patient and provider [19]. Such communication is crucial to any encounter between patient and provider as it involves not only the transfer of information but also the potential for psychosocial healing through a therapeutic relationship [19]. To learn how to effectively and empathically communicate with patients, providers receive training on techniques for “listening, explaining, questioning, counseling, and motivating” in academic and clinical settings [19 p. 13]. Provider compassion and communication styles shape experiences of anxiety and women’s opinions of their providers in the U.S. [20]. Studies conducted in other high-income countries (HICs), such as the Netherlands, show that when providers communicate warmly and empathically, these actions can reduce anxiety and increase positive expectations for treatment [21, 22]. A recent systematic review of studies conducted in multiple kinds of economies, including LMICs and HICs, found that provider communication skills were related to better health outcomes and facilitated treatment-related behaviors [23]. Research in HIC settings suggests that patient anxiety increases when general practitioners use more direct versus indirect negative messages when communicating [24]. Girgis et al. found that a consultation skills training program in Australia that aimed to improve physicians’ detection of psychological distress was associated with decreased patient anxiety [25]. Likewise, a US-based study found that empathy from physicians is associated with fewer symptoms of anxiety, improved patient ratings, and higher trust in the physician, as well as patients feeling listened to and cared about [26].

Prior research suggests that healthcare decision-making in Pakistan primarily falls to the family and medical providers in a collective decision-making process, in which the provider often has disproportionate influence [27]. Rather than considering psychosocial factors related to lifestyles, providers in Pakistan often prioritize biomedical considerations when diagnosing and interacting with patients [28]. While studies examining antenatal anxiety within the context of the patient-provider relationship in Pakistan are lacking, research from the United Kingdom has established the importance of the patient-provider relationship on women’s engagement in the antenatal care process, control over their care trajectories, and understanding of information [29, 30].

A study based in the U.S. examined the relationship between patient-provider communication, antenatal anxiety, and self-care in pregnancy [31]. Pregnant women’s perceptions of communication, collaboration, and empowerment in relationships with their midwives were associated with lower anxiety and positive health behaviors [31]. These results suggest that provider relationships can improve patient health through the provision of emotional support [31], a result echoed by other studies that also emphasize the role of patient-provider communication in influencing information exchange and the creation of a positive interpersonal relationship [29, 32]. Moreover, in the aforementioned study, lower antenatal anxiety during mid-to-late gestation mediated associations between patient-provider communication and positive health behaviors [31].

Evidence from LMICs is unclear about how perinatal anxiety is influenced by or reproduced through patient-provider relationships that are marked by differences in communication styles, sociocultural backgrounds, and treatment goals. It is possible that, in the Pakistani context, provider-patient communication could be a key factor in either mitigating or exacerbating anxiety and influencing antenatal care engagement among pregnant women. Therefore, our study expands on barriers and facilitators to patient-provider communication in a low-resource setting by exploring intersections between patient-provider communication and anxiety in pregnant women. Our aim was to understand how communication content and processes between pregnant women experiencing anxiety and antenatal providers in a public antenatal health care setting in Pakistan influence pregnant women’s experiences of anxiety.

## Data and methods

### Study setting

This study was conducted as part of qualitative research that aimed to inform the design of a psychological intervention to reduce antenatal anxiety [33]. Data were collected from providers and pregnant mothers in Rawalpindi District, Punjab Province, Pakistan. Pakistan is classified as a LMIC with a total population of 212 million [34]. The total fertility rate of Punjab Province is 3.4 live births per 1000 women, which is close to Pakistan’s total fertility rate of 3.6 live births per 1000 women as of 2017–18 [18]. Pakistan’s 2015 GINI index value of 33.5 reflects high levels of socioeconomic inequality, unemployment, and poverty [16]. Additional health indicators for Pakistan are included in Table 1.

**Table 1:** Maternal and newborn health indicators for Pakistan, 2017–18.

Health Indicator	Percentage
Antenatal care coverage for at least four visits	51.4%
Proportion of women ages 20–24 who gave birth before age 18	7.4%
Number of women aged 15–49 years with a live birth delivered in a health facility	66.2%
Proportion of births attended by skilled health personnel	69.3%
Proportion of women aged 15–49 who received postnatal care within two days after giving birth	61.9%
Infants who had their first postnatal checkup within the first two days after birth	64%

Note: All data in Table 1 is obtained from the Pakistan Demographic and Health Survey 2017–18 [18].

### Study population and sampling

All participants were recruited from a public tertiary care teaching hospital in Rawalpindi, Pakistan. This hospital provides free antenatal services to an average of 250 primarily low-income patients daily. Sample size was determined based on reaching data saturation on the interview topics related to the original study aims [35]. Providers were recruited based on their availability using purposive sampling to ensure inclusion of different types of antenatal health workers [36] (i.e., doctors, nurses, and midwives) from the study hospital’s Obstetrics and Gynecology Department. Pregnant women were approached consecutively during an antenatal visit at the outpatient clinic. Potential patient participants were then screened using an Urdu version of the Hospital Anxiety and Depression Scale (HADS) [37–39] and the Structured Clinical Interview for DSM-5 (SCID) [40]. Adult women at ≥20 weeks of pregnancy who scored ≥8 on the anxiety scale of the HADS (indicating at least mild anxiety), did not have clinical depression (measured with the SCID) or serious medical conditions, spoke Urdu, and lived within 20 kilometers of the hospital were eligible for participation. Pregnant women and providers were recruited until data saturation was reached for the main study, which resulted in the sample size that was also used in the secondary data analysis for the present study. Thirty-nine pregnant women who were screened for anxiety and depression met the inclusion criteria, out of which 19 participated. Twelve providers were approached and two declined due to time constraints. Participants were not provided incentives for participation. The research received ethical approval from the Human Development Research Foundation and the Johns Hopkins Bloomberg School of Public Health Institutional Review Board. Participants who screened positive for severe anxiety, depression, or suicidality were referred for further assessment and/or psychiatric care.

### Data collection

A team of four trained female interviewers conducted and audio-recorded in-depth interviews with 19 female patients and 10 female providers between September 2017 to August 2018. Pregnant women were interviewed by female interviewers so that they might feel more comfortable discussing matters concerning pregnancy and antenatal mental health. Interviewers were Pakistani research assistants who had graduate degrees in psychology, two years of experience conducting field interviews, and fluency in Urdu. A clinical psychologist provided a one-day training to the interviewers on how to provide informed consent, recruit participants, maintain confidentiality, and use non-stigmatized language.

Interview guides developed for patients and providers were pilot-tested after translation to Urdu and back-translation to English to ensure quality and accuracy. Topics in interview guides included, but were not limited to: causes that women attributed to their experiences of antenatal anxiety, physical and emotional symptoms of antenatal anxiety, the impact of antenatal anxiety on women’s daily functioning, and coping strategies used for antenatal anxiety. To ensure privacy, interviews were conducted in private spaces. Written informed consent was obtained from both providers and pregnant women. If a participant was unable to read or write, interviewers explained the study to the woman and to a family member who signed the consent form on her behalf. The interviews lasted for about a half hour, ranging from 14 to 75 minutes (mean = 36 minutes).

### Data analysis

We used the transcribed and English-translated data to carry out qualitative, thematic analysis of the in-depth interviews. The qualitative analysis was guided by a constructivist epistemology in which we approached coding by considering the data as a reflection of participants’ subjective realities and social constructions, whether these were unique or shared across participants [41, 42]. A team of two researchers coded patient and provider interviews with Dedoose software [43], using a combination of inductive and deductive coding detailed in a codebook [44]. To generate the codebook, both coders separately coded an initial subset of patient and provider interviews, after which they created initial codes based on emergent themes. Then, each coder coded half of all transcripts independently and met periodically to debrief regarding emerging codes and to adapt the codebook to capture commonalities and differences across the two types of participants. Memos were created primarily in the third phase of coding to document emerging overall themes. Examples of the final set of themes that emerged as relevant to patient-provider communication and antenatal anxiety included rapport between patients and providers, barriers to receiving antenatal care, financial constraints on antenatal care, time constraints affecting provision of antenatal care, and upholding of social norms by providers.

## Results

The sample included 19 pregnant married women (Table 2), ranging from 18–37 years old (mean = 26 years) and having between 5–16 years of education (mean = 10 years). Women’s number of children ranged from 1–4 with nine women being primigravida. The 10 providers included obstetrician/gynecologist physicians (*n* = 4) and non-physician antenatal providers (*n* = 6). Providers ranged in age from 24–55 years (mean = 40 years), had 1–30 years of experience (mean = 16 years), and worked an average of 7 hours per day (Table 3). We have organized the results by the following predetermined categories: pregnant women’s perspectives on communication with antenatal providers, antenatal providers’ perspectives on communication with anxious pregnant patients, integrated perspectives from patients and providers on communication during the antenatal period, and communication processes and content used by providers and their antenatal patients. All names used are pseudonyms.

**Table 2:** Characteristics of participating married pregnant women.

Participant ID (Pseudonym)	Age	Education
	(range in years)	(range in years)
M1 (Zareen)	18–24	5–9
M2 (Aleena)		
M3 (Amarah)	18–24	10–14
M4 (Sahara)		
M5 (Nooshin)	20–24	5–10
M6 (Salma)		
M7 (Zohra)		
M8 (Ahmadi)	20–24	10–14
M9 (Safeena)		
M10 (Narmin)		
M11 (Yusra)	25–29	10–14
M12 (Soofia)		
M13 (Rumaisa)		
M14 (Surayya)	25–29	15–19
M15 (Zaina)	30–34	10–14
M16 (Daneen)		
M17 (Munira)		
M18 (Shahin)		
M19 (Mehreen)	35–39	10–14

**Table 3:** Characteristics of participating providers from the study hospital’s Obstetrics and Gynecology Department.

Participant ID (Pseudonym)	Job Title	Year range of work experience
P1 (Dr. Beg)	Obstetrician/gynecologist	11–20 years
P2 (Dr. Aabid)	Obstetrician/gynecologist	21–30 years
P3 (Dr. Durrani)		
P4 (Dr. Cheema)		
P5 (Ms. Numan)	Non-physician provider	1–10 years
P6 (Ms. Ghazali)		
P7 (Ms. Hidayat)		
P8 (Ms. Jarral)		
P9 (Ms. Shaikh)	Non-physician provider	11–20 years
P10 (Ms. Izam)	Non-physician provider	21–30 years

Note: Non-physician providers included nurses and midwives.

### Pregnant women’s perspectives on communication with antenatal providers

In their accounts of antenatal anxiety, pregnant women expressed their desire for specific factors that would enhance communication with providers, particularly warmth, approachability, respect and privacy as well as trust within the patient-provider dyad.

#### Desired patient-provider communication characteristics

Pregnant women frequently mentioned the manner and tone in which providers communicate during antenatal visits as crucial to creating an open atmosphere for discussing their pregnancies, health concerns, and experiences of anxiety. Mehreen expressed a sentiment common among patients by stating *“The most important thing is to speak to the patient with love*. *You get more information”* (M19). Narmin expressed a similar sentiment saying that a good provider is *“a person who doesn’t get angry and makes you understand with love” (M10)*. Patients commonly used words such as kind, soft-spoken, approachable, and patient to describe the qualities they wanted in a provider and that they felt would facilitate the best communication. Many patients reported that without such characteristics, they would likely feel reluctant to discuss their problems or open up to providers. For instance, Salma stated that *“[providers] should have good character*, *a kind heart*, *and be soft spoken*. *Since you are asking me questions politely*, *that’s why I am answering them*, *but if you were asking me angrily*, *I might never answer even one of your questions”* (M6). Moreover, many patients cited listening skills and attentiveness as important characteristics for providers to have. As Daneen said: “*If we talk to [providers]*, *they must listen and try to understand us and we should do the same”* (M16).

A few patients were reluctant to share their non-physical concerns, such as social stressors or emotions, with providers. They viewed sharing such ‘personal’ problems as beyond the purpose of a visit to a provider; they expected instead to simply receive their check-up and leave. For example, when asked whether she would be able to talk about her personal problems openly, Narmin answered:

Narmin: “No, I would not tell [my problems].”

Interviewer: “What is the main thing that would stop you from talking about them?”

*Narmin*: *“No*, *there isn’t any*. *We come here for a checkup*, *so we have our checkup and go*.*”* (M10)

However, more patients reported a desire to be able to discuss their problems with providers as long as their providers were cooperative with them. For example, Munira said *“If someone [a woman] has a problem*, *she will share with her doctor if her doctor is cooperative*. *If the doctor is not cooperative*, *she is not going to tell them anything*. *That’s what I do*” (M17).

Many patients seemed aware that providers, especially those in public hospitals, had high caseloads and limited time but still reported expecting providers to engage thoughtfully with them. Safeena remarked on the distinction between the atmosphere of public areas in the hospital versus the quieter environment of providers’ private exam rooms:

*“The doctors should ask important questions of the patient and should not feel pressured if a patient visits them*…*Though they are in a public hospital and there are a large number of employees*, *doctors are working in a calm setting and don’t have any [major] pressures in each individual exam room*.*”* (M9)

Shahin reported a desire to be talked to patiently and to have her questions answered adequately, saying, “*Doctors should give proper treatment and if patients are having any problems*, *then listen to them in a calm manner and also give solutions to their problems related to pregnancy”* (M18). In some cases, when pregnant women anticipated that they would not be able to spend enough time with a provider or encounter a friendly provider at a public hospital, they reported visiting private hospitals instead.

Finally, patients mentioned the importance of having a private environment in which to communicate with the provider. Having family members present during medical encounters seemed to make patients less comfortable with openly communicating with their providers. As Amarah revealed to her interviewer, *“Now my sister is not here*. *That’s why I have discussed my [problems] with you”* (M3).

#### The role of respect in communication with providers

Pregnant women reported multiple examples of feeling disrespected by providers. For example, Ahmadi (M8) described an instance where she had an appointment with a provider who she felt held a grudge against her, leaving her to wait in the waiting room without explanation. Ahmadi explained that the provider eventually saw her but that she did not feel treated with respect. Moreover, many patients reported that patient-provider interactions in public hospitals tend to be less respectful than treatment at private hospitals. Surayya described how she felt nervous about being disrespected by providers at public hospitals and felt inhibited asking providers at the study hospital for their contact information, even when they treated her well:

*“Unfortunately*, *I don’t do my antenatal checkups in private hospitals and I am very scared of doctors at the [study hospital]*. *Last time when I visited the [study hospital]*, *there was a doctor who checked me well*. *I wanted to take her contact number but I was scared as another doctor sits there who scolds patients*. *And I feared that I would be disgraced in front of the other mothers there*.*”* (M14)

This sentiment was echoed by other patients, such as Mehreen who said “*[Private] health centers show very polite behavior with patients*, *which I have seen is not the case [in public hospitals]*. *I feel a lot of tension”* (M19).

The ways that providers responded to patients’ lack of knowledge appeared to be another common source of anxiety among patients. In one example, Safeena described that a provider asked her sister-in-law *“when her clothes were last ruined”* (M9) instead of asking the date of her last menstrual period. Her sister-in-law did not understand the question and was reportedly scolded by the provider. Similarly, Zohra described her fear of talking openly with her provider:

Interviewer: “You were saying you wanted to talk to the doctor about it.”

Zohra: “Yes, that is what I’m thinking whether or not I should ask the doctor about it.”

Interviewer: “Why do you feel like this is something you can’t talk to the doctor about?”

Zohra: “I feel the doctors will insult me.”

Interviewer: “So, if they do not insult you, will you be able to talk to them?”

*Zohra*: *“I need confidence*.*”* (M7)

Pregnant women commonly talked about experiences of being scolded or reproached during check-ups. Soofia explained that “*some doctors…don’t talk to you in a good way*” (M12). She continued to describe that when she went to a recent checkup, the provider asked her *“Why do you come here*, *why don’t you just stay at home*?*”*(M12), but also said that “*There are some doctors who talk to you in a good manner and ask about your problems*” (M12). In response to a question about qualities desired in a health care provider, many women discussed how providers should treat patients with respect. To illustrate, Aleena responded *“He/she should talk politely and behave well”* (M2).

#### The role of trust in communication with providers

Many patients described valuing common lived experiences that providers shared with them. For example, women mentioned that having a female provider, a married provider, or a provider who has personally experienced pregnancy increased the likelihood that the provider would be able to understand the pregnant women’s circumstances and empathize with them. Nooshin explained that *“Only a woman can understand a woman*. *When a woman talks and the other listens*, *she reads her eyes more so than her words”* (M5). Nooshin’s statement speaks to a commonly expressed belief that providers must build trust using both verbal and non-verbal communication. Moreover, another patient, Safeena, described the importance of first impressions in building a feeling of attunement:

*“[Providers] are strangers*. *We don’t know them*. *They don’t know us*. *Without knowing each other*, *you assess the other person in the first meeting*. *If the person is good to you in the first meeting*, *you feel good and then it becomes your last impression*.*”* (M9)

Participating women, such as Munira, mentioned their trust in physicians over other kinds of health providers: *“If a person is telling us what to do and he/she is not a doctor*, *people will not pay attention to him/her”* (M17). Safeena described having more trust in providers who have proper training. When asked about what kind of provider she would like to facilitate a mental health support group, she said:

*“Those who don’t have knowledge about it cannot be trained*. *It’s a profession and you have been given education for that*. *She should be a qualified teacher or professional*. *She will be taking a lot of our time so she should be qualified*.*”* (M9)

In response to same question, Munira said that “*a doctor would be the best choice for this because they are better able to understand the problems of pregnant ladies…I don’t think a professor or [other kind of] trained individual can understand this*” (M17). Patients’ trust in the training that providers received was related to their ability to guide and understand pregnant women better. Ahmadi described the characteristics that a provider ought to have in order for women to be open to discussing their experiences and sources of anxiety with them:

*“First*, *she should be married herself*, *because the one who has been through all that understands better*. *Second*, *she should be educated*, *obviously*. *I think it’s all about expertise and experience*. *If you have these things*, *you can guide others better*.*”* (M8)

### Antenatal providers’ perspectives on communication with anxious pregnant women

A major theme that emerged from provider interviews was the high frequency of conflicts and misunderstandings requiring provider-initiated negotiation. Providers also spoke about their roles as sources of comfort for pregnant women with anxiety since they often took on roles of problem solvers for health issues, financial constraints, and more, while also helping to negotiate family involvement. At the same time, providers reported that they may sometimes cause or contribute to patient anxiety, often due to their own harried demeanors and busy caseloads.

#### Conflicts or misunderstandings with pregnant women

Providers reported various types of conflicts and misunderstandings that they encountered in their work with pregnant women. Due to the limited time that providers can spend with each patient, some providers expressed an awareness that patients often feel as if providers do not fully hear them, respect them, or truly care for them. Dr. Aabid, an obstetrician/gynecologist, shared her perspective on experiences in Pakistan’s public hospitals:

*“Another contributing factor [to anxiety] can be…the attitude of healthcare providers*, *which also causes psychological disorders in patients*. *Unfortunately*, *the tertiary public care hospitals that we have here are very crowded and the one-to-one care that the patients should get is impossible*. *So*, *in this type of overburdened environment*, *many things are left unsaid by the patient*. *Many questions that she does ask are left unanswered*, *so this also creates a sense of deprivation in patients*. *They sort of feel that they are not respected or cared for*. *The adequate time and attention that they deserve are not being provided so this is a source of tension in patients*. *They want to know about their illness*, *but no one tells them in detail about the treatment*, *where they should go for tests*.*”* (P2)

Several providers reported that patients occasionally become upset with them, such as when they do not like hospital administrative procedures or medical advice that they are given. In these cases, providers reported having to control the situation, call family members, or counsel the upset patients. Some providers also described having to deal with patients’ family or financial issues. For example, one non-physician provider, Ms. Jarral, discussed how a low-income patient needed a blood test, but her husband was unemployed, describing how the patient *“was putting the responsibility for arranging the blood on the doctors”* (P8). Ms. Jarral then had to contact the pregnant women’s husband to ask him to arrange for her blood test and pay for it.

Providers also reported pregnant women’s family members as sources of conflict that they need to contend with. They described how sometimes, family members, particularly those who are older, do not understand the necessity of regular antenatal care visits and can be dismissive of providers’ suggestions. For instance, Dr. Cheema explained that when providers encourage patients to attend an antenatal check-up every month, *“our patients [often] come to us and…they say that their mother-in-law said ‘What’s the need of going to the hospital*, *you’re going for recreational activities as we have also delivered children and they are delivered at home*, *no one else delivered them for us’”* (P4). In these cases, providers talked about having to reiterate their suggestions and justify their recommendations to both patients and their family members.

#### Providers as sources of comfort

Providers demonstrated an understanding of the important role of listening in counseling patients. However, they also demonstrated an awareness of how time constraints and high caseloads detract from their ability to adequately listen to and comfort them. In the following quote, Dr. Aabid described how active, empathetic listening can comfort patients:

*“Look*, *the attitude of doctors’ matters a lot because in counseling a patient*, *the foremost important thing is listening to the patient*. *If you do listen to the patient and she thinks that you listened to her attentively and sympathetically*, *that the pain she described has infiltrated your brain and heart*, *it reduces her pain by half*. *So*, *this is why people are very dissatisfied in the extremely rushy*, *messy clinics [where no one] has the time to listen*, *despite receiving physical treatment*.*”* (P2)

Other examples of ways that providers felt they were able to provide comfort to women included restructuring their negative thought patterns, framing their concerns optimistically, and making hopeful comments. Several providers mentioned that family issues are common stressors for pregnant women and that they often console patients about their families’ attitudes and advocate for their interests. A non-physician provider said that she considered it her duty to “*console the patient not to get worried because they are already stressed by their family’s attitude*” (P5). Another provider described giving her contact information to patients so that patients could call occasionally for support.

Assuring patients of the normalcy of unpleasant symptoms that they experienced during pregnancy appeared to be one of the most common ways that providers reported calming patients’ worries. For example, one non-physician provider described telling patients that vomiting in the first trimester is common and that many mothers experience it. If that did not succeed in calming a patient, then she said that providers would counsel family members about the situation, so that family members could support the patient. As a last resort, the non-physician provider suggested that providers would prescribe anxiolytic medications.

Financial assistance was thought to play an important role in comforting patients. Providers commonly reported that in some cases where patients were stressed about their ability to afford medications or treatment, providers would work with them to find a cheaper alternative, try to convince family members to contribute funds, or even personally step in with financial help. Ms. Hidayat described one such instance that occurred after a patient gave birth:

*“One patient got some infection in her stitches*. *On the first day*, *she followed our recommendation and brought an injection of TANZO*. *The second day*, *she cried badly in the doctors’ room*. *Then*, *the doctor helped her financially and she bought an injection and got full treatment for blood loss*. *She left healthy and happy because she was feeling well*.*”* (P7)

Other strategies that providers mentioned using to comfort patients included refraining from talking about their personal problems in front of them and citing their own successful experiences with pregnancy. Providers also reported speaking to patients in their own language and/or dialect to build rapport. Ms. Izam stated “*The best thing that I feel that we do is that we converse in their own language*, *be it Urdu or Punjabi*. *This makes it easy for them to follow what we are saying*. *This makes them feel like we are one of them”* (P10). Providers frequently described mentioning faith and/or God when comforting patients. Here, Ms. Izam described how she would reframe patients’ problems and use faith to calm their anxieties:

*“Even a little compassion from us [matters]*, *like saying please don’t think like this and don’t be gloomy*. *Allah is almighty and He will fix everything for you*. *It’s just a matter of a few days*. *All problems are temporary and will come to an end*. *You will get to go home after that*.*”* (P10)

Finally, some providers spoke about teaching pregnant women that anxiety and stress can harm their health and that of their unborn baby. Providers described counseling patients that if they controlled their stress and remained content, then the chances of a successful pregnancy would increase. Moreover, Ms. Shaikh described how establishing an antenatal care routine and seeing providers regularly helps patients feel like they are being taken care of. She said, “*When a patient has their regular checkups*, *gets their laboratory tests*, *ultrasounds*, *and CT scans done*, *then they feel that the doctors are good to them*, *they are being taken care of*. *The patients come out of that cycle of anxiety this way”* (P9). As mentioned by a physician, pregnant women typically want to know if everything is alright with their health and with their baby, so reassuring them with these questions at each antenatal care visit is important. Another physician described how she views considerate communication and reassurance as antidotes to anxiety:

*“If the patient talks to you and you listen attentively*, *then patients mostly say*, *‘Doctor*, *I have been halfway treated by coming to you*.*’ So*, *I don’t think that they get physically well by just visiting the doctor*. *Physical disease continues to exist*. *It is their mental strain that lessens from just meeting the doctor*, *through examination [and] reassurance*.*”* (P2)

#### Providers as sources of stress

Many providers gave accounts of how they felt they contributed to patient stress by using harsh words with patients. Ms. Ghazali explained:

*“If someone talks to you rudely*, *it makes sense that you would get angry*, *and when they are in this condition (pregnancy) then it is not permissible to make them angry*. *When they get angry*, *their blood pressure gets high and when their blood pressure rises it also affects them and it is harmful for the baby and that happens just because of our behavior*.*”* (P6)

Many providers admitted that due to organizational challenges in Pakistan’s public hospitals (being overburdened with job responsibilities and having limited time for individual patients) they sometimes spoke to patients harshly, possibly inhibiting open communication. For example, Ms. Jarral spoke about how providers treat mothers during labor and delivery.

*“The behavior of the doctor also influences the patient at the time of delivery*. *There was a doctor in Lahore who was a gynecologist…[who] used to go abroad for her delivery*. *Because there is a different way of dealing with pregnant women [abroad]*, *as the midwives sit beside the mother*, *holding her hands during the labor pain and here*, *you know*, *doctors deal with delivery very differently and sometimes their behavior is not appropriate*.*”* (P8)

To solve the problem of providers treating patients rudely, another non-physician provider suggested that increasing staff and beds in the hospital wards would enable providers to give more individual attention to patients and lessen their use of harsh language, thereby impacting patients’ anxiety levels and even physical health. Ms. Izam stated that *“Our cooperation is very essential for [patients] to be stable*. *Otherwise*, *they can end up with high blood pressure”* (P10). According to Ms. Ghazali, sometimes the public hospital environment is filled with so much tension that patients *“run away”* (P6). In particular, she believed that after hearing screaming from the labor room and witnessing the occasionally inappropriate behaviors of physicians and other staff members, patients sometimes decide to get care at a private hospital instead.

### Integrated perspectives from patients and providers on communication during the antenatal period

Patients and provider accounts revealed differences in socioeconomic status (SES) and sociocultural norms between the groups. These differences appeared to shape communication, as well as pregnant women’s care-seeking behaviors and providers’ personal views of them. Patients and providers often seemed to agree on how SES differences affected patients’ abilities to access care but did not always agree on how their own sociocultural beliefs and their perceptions of each other’s sociocultural beliefs influenced patient anxiety and health behaviors.

#### Socioeconomic influences on patient-provider communication and antenatal health care interactions

The low-resource setting of the study hospital seemed to govern patient-provider communication to a large degree and the SES gap between patients and providers at the hospital was noted by both groups. Patients expressed views that providers had comfortable lives, even if they had packed caseloads. One pregnant women went as far as to express that a good quality provider *“should think others are better than themselves”* (M3). Meanwhile, providers consistently took into consideration patients’ financial constraints when prescribing medication, recommending treatments, and suggesting lifestyle changes. Many providers described how seemingly simple recommendations must only be offered after considering their financial ramifications as demonstrated in this statement from Dr. Beg:

*“Affordability is a major issue for our class [of low-income patients]…See*, *if we tell the patient to do a protein diet*, *what’s the price of mutton or beef nowadays*? *If you ask the patient about how many times they cook it in a month*, *then [it is] maybe once for the lower class*. *Then*, *what’s the number of members left in the house*, *after it gets distributed once*?*”* (P1)

When prescribing medications, providers described needing to consider how long the patient might be able to afford a long-term medication. Similarly, when advising patients to obtain diagnostic testing, they had to consider where such testing would be offered. For example, if the testing was only available in a private hospital, then the patient might not be able to afford it. Even highly discounted testing in public hospitals might pose a financial issue for many patients. Dr. Beg said that *“if the ultrasound is being done in the [public] hospital*, *then it’s minimally charged*. *Extremely minimal*. *Many times*, *maybe the patient is still unable to afford it”* (P1). In high-risk pregnancies, the number of visits and tests needed can be high, and providers described having to be cognizant of how high-risk patients’ financial limitations may affect their treatment and health outcomes.

Ms. Shaikh discussed how pregnant women often asked her about costs for tests that she recommended to them and would only act on her recommendations after ensuring affordability:

*“Mostly women do not share [their finances] with us openly*, *but we can feel this thing from their attitude*. *For instance*, *when we advise women to get their laboratory test done then they ask us about the charges of each test*. *Thus*, *we can access from their facial expression that it’s difficult for them to afford such tests*. *If a person can afford it*, *they will never ask you this information*. *[Pregnant women] actually first find out about their expense*, *then go for tests*.*”* (P9)

While she said that most patients reveal low SES through indirect questions about cost, Ms. Shaikh also mentioned that *“There are some patients who are assertive; they directly tell the doctor that they cannot afford the tests”* (P9).

Pregnant women seemed aware of their own low SES and the fact that public hospitals tend to treat low SES patients, often comparing their experiences at public hospitals to what they perceived as higher-quality treatment for higher SES patients who could afford to attend private hospitals. Mehreen exhorted high SES providers at public hospitals to treat low SES patients with more respect regardless of their financial constraints:

*“If the provider is well educated and trained*, *what use is [their education and training] if they treat the patient with disrespect*? *This [situation] is especially true of government hospitals*. *Private hospitals take fees; they give respect to pregnant women*. *Poor people have no more funds to go to a private hospital*. *They cannot afford to*. *I say to government hospitals*, *you should speak to [poor patients] with love*. *I heard there was a hospital in Abbottabad that had open beds*, *believe me*, *but a woman was left to lay on the floor with her IV line*. *I say*, *government hospitals should please*, *please treat patients well*. *Speak to them with love*. *You can’t imagine how endless their prayers for you will be*.*”* (M19)

Several pregnant women reported feeling a sense of injustice and tension at how high SES providers treated them and believed that providers at for-fee health facilities were more courteous to their patients because they could pay for treatment. Meanwhile, providers described how patient anxiety about their limited financial resources was shown in the verbal and non-verbal ways that patients inquired about treatment costs. Some providers reported attempting to integrate treatment costs and financial constraints into their discussions with patients.

#### Sociocultural influences on communication

Providers described several sociocultural differences that they encountered between patients and themselves. Firstly, according to providers, patients tended to describe their experiences of ‘anxiety’ in highly somaticized language that did not necessarily frame anxiety as a mental or emotional disorder. Many providers explained that patients described their anxiety in discrete physical terms, such as “*sar me dard”* (headache), *“saas phoolti hai”* (breathlessness), and “*bechayni”* (restlessness). As such, some providers reported relying on a combination of patients’ body language, medical history, and description of symptoms to diagnose them with anxiety. Ms. Hidayat told how anxious pregnant women “*mostly are unsure of what is happening around them and say that can’t do anything and mostly use facial expressions to show their anxiety”* (P7). However, even when they attempted to reduce patients’ anxieties, such as by normalizing unpleasant symptoms, educating patients about anxiety or prescribing anxiety medication, they felt that patients might not understand that they were experiencing a mental health condition instead of its resulting physical symptoms. In this example, Dr. Durrani described how patients tend to lack insight into anxiety as a condition that can cause a wide range of psychosomatic symptoms and can be effectively treated by a mental health professional:

*“Women have no idea they have anxiety*, *they don’t go to the doctor*, *they do not understand about treatment*. *They should be checked by a psychologist or psychiatrist*. *They do not understand this; they take this as a physical illness*. *This is very important [to understand] and this is not [physical] illness*.*”* (P3)

When asked what stops women from reporting that they experience anxiety, she said that patients *“have no realization and…they feel it is not a big problem to share [their anxieties] and no benefit to share this*. *But this is very important*, *if they have no insight*, *then how will they recover*?*”* (P3). In contrast, several patients contested the notion that healthcare is the answer to reducing their symptoms of anxiety. Instead, they emphasized the importance of the social environment in shaping a woman’s mental and emotional state. Safeena said that “*neither diet nor blood are helpful for women’s health*, *but only atmosphere matters”* (M9).

As another emerging theme, many patients and providers commented on how social norms governing women’s roles in their households and families influenced their experiences of anxiety and their interactions with the antenatal healthcare system. These social norms often included a degree of subservience to husbands and elder family members. For example, in answering a question about how spousal conflict affects pregnant women, Dr. Durrani illustrated possibly stigmatized beliefs towards lower- to middle-SES women when she stated:

*“I think our women accept these things [husbands not being cooperative]*. *Basically*, *these women are from the lower-middle class*. *They have no authority and control over themselves and their husbands*.*”* (P3)

In another example, Surayya described how whenever she talked to her husband about a health concern, he would minimize it by telling her not to worry and that he would take care of her concerns himself. She went on to express how she feels that antenatal providers should communicate with the woman and her husband simultaneously:

*“When the pregnant mothers come to the doctor*, *[the doctor] should do all antenatal checkups in front of their husbands*, *so they know the condition of their wives and know what necessary tests are required…there is no use to talk only to women*, *but husbands should also be present because they have to spend a long time together*.*”* (M14)

Several providers reported potentially stigmatizing or stereotyped views of women, especially those of particular ethnic minority groups or low SES backgrounds. These views appeared to shape their communication with and recommendations to anxious women in antenatal care encounters. For example, some providers either assumed that their female patients were responsible for housework or even actively endorsed traditional gender norms, such as in this example from Dr. Aabid:

*“To physically look after a child*, *feeding them milk*, *looking after them psychologically*, *making them a good human being*. *This task should not be called a burden but is a responsibility that has to fall on the mother*.*”* (P2)

Other providers framed low SES women’s lives as governed largely by housework and repeated pregnancies, suggesting that they sometimes take advantage of pregnancy strategically in order to secure certain privileges. For instance, Dr. Beg displayed possibly prejudiced views against the Pathani ethnic group (a small minority group prevalent in Western Pakistan [39]), by saying how some women *“get pregnant again and again because they know they will be given some extra care during this time*, *regarding the diet or rest point of view*. *This is very common among Pathans*, *as they consider themselves very important”* (P1). A few providers expressed the view that low-income pregnant women have cultural views grounded in ignorance. For instance, in response to a question about how women who have had prior miscarriages cope with their current pregnancies, Ms. Jarral appeared to shift blame onto them when she said:

*“Some of the women wear amulets and doing all this is such a waste of time*. *Many women get benefit from it and many women do not benefit from it…Women themselves create their own problems*. *They can overcome their problems themselves…If you ask about me personally*, *the condition of my home is different and my lifestyle is different*.*”* (P8)

Such sentiments containing elements of stigma and/or prejudice were much more common among providers; SES- and sociocultural prejudices were not commonly expressed by patients towards providers. While providers did not often report directly conveying stereotyped views about patients to them, some providers mentioned making decisions about how to deal with women who communicated cultural beliefs that they did not agree with. For instance, Dr. Beg discussed how she would ignore it when patients mentioned that older generation family members held cultural beliefs against visiting an antenatal doctor regularly during pregnancy.

*“It’s a very normal sentence that we hear from everyone and we don’t pay heed because what answer do we have to this*: *a mother-in-law who gave birth to eight children at home and never went to the doctor and the daughter-in-law says ‘I have to go to the doctor*.*’”* (P1)

Meanwhile, some patients lacked confidence in providers’ abilities to help them with their anxiety. For example, when asked how providers could help her to deal with her apprehensions surrounding delivery and surgery, Zohra said “*doctors cannot do anything*. *It’s all in Allah’s hands*. *[The apprehension] will go away on its own*” (M7). Another patient, Safeena (M9), wondered out loud whether her mother was correct that it would be better for her not to verbalize her fearful thoughts, such as anxiety about her fetus’s movement, in order to protect against the evil eye. Women also described how lack of family cooperation with healthcare providers’ advice could arise from differences in cultural norms around how care should be received by women and how women should interact with providers. Some women mentioned that their families and/or communities viewed the hospital as a place to quickly receive medicine or treatment. Yusra described community members’ general attitude towards mental healthcare professionals administering treatment for anxiety at hospitals as *“they don’t do any work*, *they are just willing to corrupt the mind of women…‘You went to get medicines*, *get medicines and come back’”* (R2). She said that this attitude is prevalent in rural areas, but that it happens in most cities too. Illustrating a similar point, some providers described how many patients’ families believe that they must only visit the hospital for an antenatal checkup at the conclusion of a woman’s pregnancy. One non-physician provider commented on the role of family members in shaping women’s antenatal care experience:

*“There are issues*, *such as when a family*, *after their first visit to the hospital*, *get their antenatal card issued and then they don’t come further for regular checkups…the mother-in-law thinks that delivering a baby is not a big deal*. *Husbands are careless in taking their wives to hospital for checkup*. *When a woman in this condition visits a hospital in the last trimester*, *then we have no record of her checkups*, *lab tests*, *history*, *blood pressure*, *sugar*, *hemoglobin*. *We cannot recommend necessary precautionary measures to her*. *We are close to square one*. *Sometimes a woman comes with heavy bleeding and her family members are not cooperative*. *In this situation*, *her family members avoid giving blood to her and put the responsibilities [of giving blood] to other family members or in-laws*. *In this situation*, *only a woman suffers*.*”* (P5)

Dr. Beg, mentioned how pregnant women face a social expectation of taking care of men first, then their children, and lastly themselves. She said that when she tells her patients to follow a certain diet, for example, she is cognizant of this norm and knows that the women might not *“feel like [she] can say it when she goes home at all”* (P1). However, many patients also spoke to differences in the ways that families influence women’s experiences of antenatal care with some families reportedly having no problems with women making their own healthcare decisions and other families granting permission to women to receive healthcare and interact with providers as they saw fit.

### Communication processes and content used by providers and their antenatal patients

Our findings point toward multiple challenges faced by Pakistani antenatal care providers in deciding what and how to communicate to pregnant women, particularly those experiencing symptoms of anxiety, in the public healthcare setting. Providers described having to remain conscious of time constraints and the needs of their entire caseload, while also paying attention to and building rapport with individual patients. They reported the need to account for various factors outside the healthcare context that influenced how women would respond to treatment recommendations, such as SES, family involvement, and health beliefs. Meanwhile, patients and providers both disclosed holding stereotyped views about each other’s sociocultural backgrounds, some of which appeared to shape how they communicated and interacted in the antenatal healthcare encounter. The key factors that influenced patient-provider communication between antenatal providers and pregnant women included both those related to the communication processes (e.g. perceptions of each other, tone, demeanor, etc.) and the content of that communication (e.g. reporting of symptoms, normalization of fears, discussions of psychosocial factors related to treatment, etc.) that jointly influenced patients’ experiences of anxiety (Table 4).

**Table 4:** Factors influencing communication processes and content reported by participating pregnant women and antenatal providers.

	Communication Processes	Communication Content
**Patients**	Degree of open sharing	Physical symptoms
	Amount of compliance with providers’ recommendations	Mental/emotional symptoms
	Level of trust in providers’ expertise	Coping strategies for anxiety
	Perceptions of providers’ lived experiences as related to the treatment process (i.e. pregnancy, marriage, etc.)	Behavioral factors related to pregnancy (i.e. dietary habits)
		Concerns regarding family members’ opinions of or influence over treatment
		Concerns regarding finances
**Providers**	Degree of respect given to patients	Financial evaluation of treatment routes or medication options
	Level of empathy toward the patients	Normalization of patient fears
	Amount of hurriedness	Mediation of patient-family relationships
	Perceptions of patients’ socioeconomic or sociocultural backgrounds	Spiritual or religious contextualization

## Discussion

This is the first qualitative investigation of patient-provider communication and antenatal anxiety in the public healthcare setting of Pakistan. Our study bolsters existing literature on patients’ desire for compassion and respect from their providers [21, 22, 45] while adding to the discourse around how antenatal providers care for low-SES patients within resource constrained environments. Our major findings center on pregnant women’s desire for compassion, respect, and trust in communication with antenatal providers. Our findings from antenatal providers corroborated the importance of empathy and compassion in communication with pregnant patients, but also highlighted how sociocultural differences between patients and providers, financial constraints, and time constraints can lead to increased patient anxiety. Overall, the low-resource setting of the study hospital emerged as a major barrier to effective patient-provider communication, potentially leading to reduced courtesy, compassion, and trust in the patient-provider dyad.

Our primary results suggest that pregnant women with anxiety seek warmth from antenatal providers and find comfort in being able to relate to providers, establish trust with them, and engage in empathic and respectful communication with them. These results align with others’ findings that providers’ tone of voice and warmth can reduce patient anxiety [21, 22]. They underscore the healing and therapeutic role that even non-mental health providers can have for anxious patients [19, 26] and the potential for caring patient-provider communication to reduce pregnant women’s anxiety and increase positive expectations for treatment [21, 22]. Our results also appear consistent with Nicoloro-Santa Barbara et al.’s (2017) findings that a patient-provider relationship marked by strong communication and collaboration can reduce patient anxiety and increase advantageous health behaviors [31].

These results stand in contrast with Jalil et al.’s (2017) study, which found that most diabetes patients in a public clinic in Punjab Province did not mind providers communicating with them disrespectfully and even considered physicians as superior to themselves [46]. Patients in that study tended to connect their satisfaction with providers to relief from pain and successful physical health outcomes instead of providers’ behavior or demeanor [46], but women in our study overwhelmingly connected providers’ communication style to their satisfaction with antenatal care encounters. One explanation for this contradiction could be that caring communication holds greater value during the antenatal period since the majority of women do not have pain or illness, as opposed to when patients deal with other health conditions where communication may feel secondary to physical discomfort. It has been suggested in the US that due to the intimate and memorable nature of the perinatal period, patients may form closer relationships with obstetrician/gynecologists as compared to other kinds of physicians [47]. One study found that the odds of patient satisfaction resulting from obstetrician/gynecologist providers’ caring and friendly attitude were three times higher than other kinds of specialists [47]. Others have suggested that the antenatal context in LMICs may be different from sick outpatient care because of low SES patients’ perceptions that antenatal care has low utility and a high opportunity cost for time and effort given that it is a preventative care service [48]. This assertion is consistent with our findings that many patients reported that their family members did not believe that obtaining antenatal care was worth the trip or expenses and that some providers reported that they had to justify the importance of consistent antenatal care.

A strength of our study is its inclusion of provider voices in addition to patient perspectives on communication in the antenatal setting. In contrast to Nadir et al.’s (2018) study, which suggests that providers in Pakistan focus on biomedical factors in patients’ illness experiences instead of psychosocial factors [28], providers in our sample expressed a keen awareness of the importance of demonstrating empathy in their communication with pregnant women and helping to alleviate their anxieties through acknowledging and addressing psychosocial stressors. Our findings also differ from prior studies that describe conversations between providers and patients in Pakistan as one-sided and authoritarian [27, 49] by revealing various ways in which provider communication in the antenatal care setting takes into account patients’ social contexts.

Antenatal providers in our study recognized the potential role of patient-provider communication in exacerbating or relieving anxieties in pregnant women. External factors constraining the ability for providers to provide empathy or address anxieties in the antenatal visit included time constraints, high caseloads, and family members’ involvement. The factors that emerged from our analysis as key contextual constraints on patient-provider communication are consistent with Feldman-Stewart, Brundage, and Tishelman’s [50] model of patient-provider communication, which posits that circumstances of the clinical encounter can cause patients’ and providers’ goals to be compromised, adjusted, or never reached.

Despite a shared awareness of providers’ external constraints, pregnant women in our study reported an ability to trust and open up to antenatal providers who had common lived experiences with them and ample medical experience and education. Although there is a dearth of research on the role of trust in improving communication between antenatal providers and pregnant women in LMICs, one review study of cancer patients in HICs found that when oncologists communicate their expertise, efficiency, and technical skills, patients tend to trust them more [51–55], a finding consistent with our results. The same study also found that cancer patients’ trust in their physicians decreased their fears, worries, and perceived risk of illness [51]. Moreover, research on cancer patients has shown that they identify trust as a prerequisite and facilitator of open communication and satisfaction with communication [51, 56, 57]. Our results coincide with the aforementioned studies by demonstrating that pregnant women view trust as an important element of communication that reduces their experiences of anxiety.

Hospital factors limiting the quality of antenatal provider-patient communication were perceived by patients and providers as particularly relevant to public healthcare facilities as compared to private healthcare facilities. Patients portrayed private hospital providers as giving more individualized, higher-quality, and more respectful care and attention to their patients, while the public antenatal care providers in our sample described financial and time constraints as endemic to the public health sector. This qualitative finding is consistent with Hassan and Rehman’s (2011) study that found provider workload to be heavier and patient-provider communication quality to be poorer in public hospitals [58]. Multiple studies, mainly from HICs, have found that patients at larger hospitals, teaching hospitals, and hospitals with decreased privacy tend to have decreased satisfaction [47, 59, 60]. In line with these findings, it makes sense that pregnant women’s perceptions of the large teaching study hospital setting may have heightened their anxieties since they have less control of their environment in this setting as compared to a private, smaller, or inpatient care setting [47]. Pregnant women in our sample expressed worries about obtaining antenatal care from public healthcare settings based on the expectation that providers would treat them discourteously or hurriedly, an expectation that both patients and providers identified as an important barrier to opening up about anxiety symptoms or other stressors when receiving antenatal care. Moreover, several patients’ reported that they felt their low SES negatively influenced how antenatal providers in public hospitals communicated with them, a result that aligns with Irfan and Ijaz’s (2011) assertion that private hospitals in Pakistan devote more attention to meeting patient needs since they rely on higher SES patients’ patronage to remain profitable [61]. Other studies, including one on antenatal care satisfaction in an LMIC setting [48], have found that patients value provider communication that does not stigmatize their low SES [62]. More research is warranted to further characterize differences between the public antenatal care context, such as the setting of this study, and the private sector to determine how perceived communication quality differs and which factors potentially contribute to differences in communication between pregnant women and antenatal care providers in these settings.

While this study focuses on interpersonal dynamics between provider and patient, the complex structural and cultural forces that may shape communication in the antenatal care encounter cannot be overlooked. Although both patients and providers in our sample were Pakistani, differences in SES, ethnic origin, and sub-cultural understandings emerged in their respective interviews. Stereotyped perceptions of low SES individuals or of particular ethnic minority groups may influence communication in the antenatal care environment, possibly leading to discriminatory procedures and interactions in a concept that Knight (2020) calls ‘enacted stigma’ [63–66]. While these perceptions may not be verbally expressed, they still constitute internal belief systems, as labelled in Feldman-Stewart et al.’s communication framework, can be communicated non-verbally and/or unintentionally [50], and have the potential to change how messages are conveyed and received. Such perceptions can also influence providers’ decision-making processes for diagnosis, treatment, and case management [67]. Future research should delve deeper into the relationship between stereotypes held by antenatal providers and pregnant women’s experiences of anxiety.

Some limitations of our study include a smaller number of provider interviews compared to the number of interviews with pregnant women and the reliance on secondary analysis of data collected at one facility with the purpose of informing the design of a preventive intervention targeting antenatal anxiety. Although saturation was reached on the topics of antenatal anxiety sources, manifestations, and coping strategies, we may not have reached saturation on all issues relevant to the patient-provider relationship or communication in the antenatal care setting. However, despite the smaller number of provider interviews, they tended to be richer than the average patient interview. Due to logistical constraints, primary data collection was completed at a single public hospital in Pakistan. Future studies could consider collecting data from multiple public hospitals across several regions in Pakistan as well as from private hospitals in South Asia. The inclusion of only patients with current anxiety could be seen as a limitation, but it also allowed for an in-depth analysis on the theme of anxiety in relation to patient-provider communication which, to our knowledge, has not been widely studied. Therefore, a strength of this study was its focus on the experience of pregnant women with at least mild anxiety and its exploration of the role of patient-provider communication on these patients’ experiences.

Other study strengths include our use of an iterative coding method [44], a combination of inductive and deductive analytic methods, and an inclusion of both patients and multiple types of providers. An iterative coding method allowed for an adaptive coding scheme, where each phase of coding built upon the results of the prior phases [44]. The use of two coders reduced the potential for bias in the analysis. Lastly, since the sample of providers included physicians and non-physician providers (i.e. nurses and midwives) with various levels of experience, the study’s results speak to the multiplicity of patient interactions with various kinds of providers at public hospitals in Pakistan.

Our results contribute to literature on patient-provider communication and mental health outcomes in LMICs. The findings provide context as to how anxiety is influenced by and reproduced through patient-provider relationships that are marked by differences in styles of communication, sociocultural backgrounds, and treatment goals. As such, the findings can be used to inform mental health interventions in low-resource settings that target both patients and providers. For example, interventions targeting patient-provider communication in the antenatal healthcare setting could encourage the use of empathic listening among providers and active participation by pregnant women by employing training to providers on how to incorporate these elements in the short timeframe of an antenatal care visit [68]. Finally, paired communication training could simultaneously target providers and patients in order to improve patient-centered communication [69].

## Conclusion

This study reveals the existence of multiple fault lines within patient-provider communication in Pakistan’s public antenatal care setting that should continue to be explored in future research. Specifically, we found that high patient caseloads, time and financial constraints, family involvement, and socioeconomic and sociocultural stigma adversely influenced patient-provider communication. Our results suggest that tangible resource constraints in the study hospital, such as a high patient-provider ratio, pregnant women’s low SES, and providers’ limited time translated into interpersonal constraints, including reduced individual attention, diminished empathy, and less courteous tones. These interpersonal constraints appeared to contribute to pregnant women’s anxieties. Future studies on communication dynamics in antenatal settings could be extended to include private hospitals in Pakistan as well as to explore the role of sociocultural stereotypes and SES differences in influencing patient-provider communication in low-resource antenatal settings in South Asia. Such research could help to shed more light on the complex determinants of pregnant patients’ anxiety and to enhance maternal and child health outcomes in low-resource settings.
